# Clinical Effectiveness of the Queen Square Intensive Comprehensive Aphasia Service for Patients With Poststroke Aphasia

**DOI:** 10.1161/STROKEAHA.120.033837

**Published:** 2021-06-10

**Authors:** Alexander P. Leff, Sarah Nightingale, Beth Gooding, Jean Rutter, Nicola Craven, Makena Peart, Alice Dunstan, Amy Sherman, Andrew Paget, Morvwen Duncan, Jonathan Davidson, Naveen Kumar, Claire Farrington-Douglas, Camille Julien, Jennifer T. Crinion

**Affiliations:** 1 UCL Queen Square Institute of Neurology (A.P.L., S.N.), University College London, United Kingdom.; 2 Institute of Cognitive Neuroscience (J.T.C.), University College London, United Kingdom.; 3 University College London Hospitals NHS Trust, United Kingdom (A.P.L., B.G., A.S., A.P., J.D., N.K., C.J.).; 4 Linguistic Resolutions, United Kingdom (J.R., C.F.-D.).; 5 Royal London Hospital, Barts Health NHS Trust, United Kingdom (N.C.).; 6 City, University of London, United Kingdom (M.P.).; 7 Homerton University Hospital NHS Foundation Trust, United Kingdom (A.D.).; 8 Great Ormond Street Hospital for Children NHS Foundation Trust, United Kingdom (M.D.).

**Keywords:** aphasia, communication, documentation, quality of life, speech

## Abstract

Poststroke aphasia has a major impact on peoples’ quality of life. Speech and language therapy interventions work, especially in high doses, but these doses are rarely achieved outside of research studies. Intensive Comprehensive Aphasia Programs (ICAPs) are an option to deliver high doses of therapy to people with aphasia over a short period of time.

**Methods:**

Forty-six people with aphasia in the chronic stage poststroke completed the ICAP over a 3-week period, attending for 15 days and averaging 6 hours of therapy per day. Outcome measures included the Comprehensive Aphasia Test, an impairment-based test of the 4 main domains of language (speaking, writing, auditory comprehension, and reading) which was measured at 3 time points (baseline, immediately posttreatment at 3 weeks and follow-up at 12-week post-ICAP); and, the Communicative Effectiveness Index, a carer-reported measure of functional communication skills collected at baseline and 12 weeks.

**Results:**

A 2-way repeated measures multivariate ANOVA was conducted. We found a significant domain-by-time interaction, *F*=12.7, *P*<0.0005, indicating that the ICAP improved people with aphasia’s language scores across all 4 domains, with the largest gains in speaking (Cohen’s *d*=1.3). All gains were maintained or significantly improved further at 12-week post-ICAP. Importantly, patients’ functional communication, as indexed by changes on the Communicative Effectiveness Index, also significantly improved at 12-week post-ICAP, *t*=5.4, *P*<0.0005, also with a large effect size (Cohen’s *d*=0.9).

**Conclusions:**

People with aphasia who participated in the Queen Square ICAP made large and clinically meaningful gains on both impairment-based and functional measures of language. Gains were sustained and in some cases improved further over the subsequent 12 weeks.

## Abstract 2

Supplemental Digital Content is available in the text.

Aphasia (an acquired disorder of language) has the greatest negative impact on quality of life of any medical condition,^1^ but how best to treat it? Systematic reviews of aphasia therapy support the hypothesis that the greater the number of therapy hours, the larger the gains in language recovery in the chronic stage poststroke.^2,3^ People with aphasia (PWA) clearly benefit if they have on average over 100 hours of therapy. This target seems to be out-of-reach for most community-based health care services, including the UK NHS where a PWA might reasonably expect ≈8 sessions in total.^4^ An efficient way to deliver the high doses required for chronic PWA is via an Intensive Comprehensive Aphasia Program (ICAP). Several have been trialled, mostly in the context of a research programme^5^ and never before in the NHS. ICAPs provide high-intensity therapy at a minimum of 3 hours per day over at least 2 weeks; use a range of formats and treatment approaches including group and individual therapy; target impairment, activity and participation levels of language and communication functioning in line with the World Health Organization International Classification of Functioning framework; include education support for the individual and for families; and have a defined start and end date, with a set cohort of individuals entering and leaving the program together. Our ICAP contained all of these elements and is the first to have neuropsychological input for both assessment of PWA and therapy for both PWA and their families. Cohorts of 3 to 4 PWA attended the ICAP as day cases (nonresidential). Attendance was required for 7 hours per day, 5 days per week, for 3 weeks, much like the intensive upper-limb program that also runs at the National Hospital for Neurology and Neurosurgery.^6^ The treating team of clinicians comprised speech and language therapists, speech and language therapist assistants, neuropsychologists, and a neurologist. See Materials in the Data Supplement for the full Template for Intervention Description and Replication (TIDieR) tool. This reporting guideline is designed to improve the documentation and implementation of clinical interventions. Post 3-week intervention, some patients were referred on to appropriate community services but did not receive any more therapy from the ICAP team.

The service was funded for 2 years, and we just completed the first one (12 cohorts of patients) when coronavirus disease 2019 (COVID-19) caused it to pause. The ICAP is registered as a service audit (National Hospital for Neurology and Neurosurgery: Ref 61-202021-CA) and as such, the board have waived the need for patient consent.

## Methods

Forty-seven participants took part, with one dropping out after a week. Summary demographic details are as follows (median [interquartile range]): age, (51 years [45–60]); months postonset, (29 [18–53]); gender, 32 male; cause of stroke: left middle cerebral artery infarct, 38; right middle cerebral artery infarct, 1; left-sided hemorrhage, 7.

Here we report data from the 2 main outcome measures collected. (1) A language impairment-based measure, the Comprehensive Aphasia Test^7^ from which we generated a score for each of the 4 main domains: spoken picture description (range, 9–87), written picture description (9–66), comprehension of spoken language (0–64), and comprehension of written language (0–62). (2) A functional communication measure, the Communicative Effectiveness Index (CETI), which is scored by the PWA’s carer/relative/friend.^8^ We also collected data on PWA’s individual goals, as well as mood and quality of life outcomes for PWA and their carers. These data will be reported in subsequent articles. The data that support the findings of this study are available from the corresponding author upon reasonable request.

SPSS software was used for all analyses. Statistical significance was set at *P*<0.05 for all planned tests with a Bonferroni correction applied to all post hoc tests (*P*<0.0125). Greenhouse-Geisser estimates were used where sphericity was violated. We tested the following 3 hypotheses:

### Does the ICAP Significantly Improve Language Ability in PWA Across the 4 Language Domains?

A 2-way repeated measures multivariate ANOVA was conducted. Language domain (multiple dependent variable) had 4 levels (speaking, writing, auditory comprehension, and reading) and time had 3 (baseline, 3-week [immediately post-ICAP] and 12-week post-ICAP). We tested for an interaction between factors and explored these with post hoc tests. We also tested for significant changes between 3-week and 12-week scores. Because repeated measures tests reject any subjects with even a single data point missing, the analysis was performed on data from 36/46 patients. 20/552 data points were missing (3.6%). Two PWA were uncontactable at 12 weeks (COVID-19), and the majority of the remaining missing data was due to PWA not wishing to attempt written picture description.

### Do Common Demographic Variables Affect the ICAP Language Outcomes?

A repeated measures multivariate ANCOVA was conducted to determine the impact of 3 demographic variables (age, months poststroke, and gender converted to categorical variables) on the main analysis outlined above.

### Does the ICAP Significantly Improve Language Function for PWA?

The CETI was collected at 2 time points, baseline and at 12-week post-ICAP, so a paired *t* test was employed. Not all PWA had a carer/relative/friend, so this analysis was performed on data from 36/46 patients.

#### Effect Sizes

We quantified change using a standardized measure (Cohen’s *d* for repeated measures data^9^) and an unstandardized measure. The latter is a calculation of how much of the gap between the PWA’s baseline score and the normal performance threshold has been closed: the back 2 normal percentage.^10^ For the Comprehensive Aphasia Test, this threshold is the 95% cutoff derived from nonaphasic stroke patients; for the CETI it is a score of 88.

## Results

### Hypothesis 1 (Domain-by-Time Interaction on the Comprehensive Aphasia Test Data)

**Figure 1.**
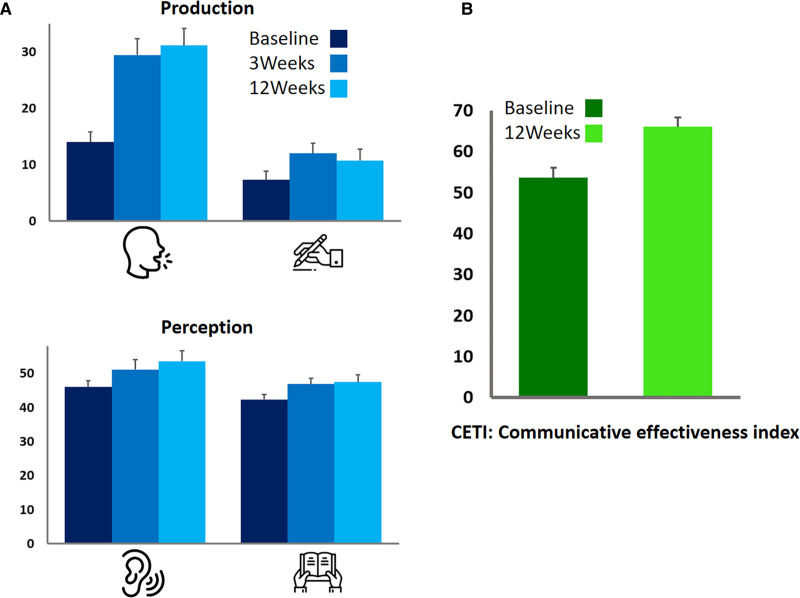
**Impairment-based outcomes (CAT). A**, Bar charts showing average language scores and (between subject) SEM error bars for speech production (top) and speech perception (bottom) domains, across the 3 time points (baseline, dark blue; 3 weeks [immediately post-Intensive Comprehensive Aphasia Program (ICAP)], mid blue; and 12 weeks, light blue). **B**, Average Communicative Effectiveness Index (CETI) scores with (between subject) SEM bars at baseline (dark green) and 12 wk post-ICAP time point (light green).

There was an interaction between language domain and therapy time *F*(2.9,100.3)=12.7, *P*<0.0005, with monotonic increases in scores over time, with one exception (Figure 1A). Planned sub-analyses of each language domain scores also demonstrated significant improvements over time: speaking *F*(2,70)=36.3, *P*<0.0005; writing *F*(2,70)=16.9, *P*=0.001, auditory comprehension *F*(1.5,52.6)=10.2, *P*=0.001; and reading *F*(1.7,58.2)=17.4, *P*=0.002. Paired post hoc comparisons demonstrated that the domain-by-time interaction was driven by speaking > the other 3 domains (versus writing, auditory comprehension, and reading, *Ps* all <0.0005). Lastly, 2 of the domains showed small but significant improvements between 3-week and 12-week timepoints: speaking *t*(43)=2.2, *P*<0.034 and auditory comprehension *t*(41)=2.3, *P*<0.028.

### Hypothesis 2 (Are Therapy Effects Explained by Demographic Factors?)

No. The domain-by-time interaction remained significant when age, gender, and time since stroke were added into the repeated measures multivariate ANCOVA: *F*(1.4,20.2)=12.6, *P*=0.001.

### Hypothesis 3 (Does the CETI Change Between Baseline and 12 Weeks?)

A paired *t* test demonstrated a significant effect of therapy, *t*(35)=5.4, *P*<0.0005, with the 12-week CETI scores higher by an average of 12.4 points compared with Baseline (Figure 1B).

### Effect Sizes

**Figure 2.**
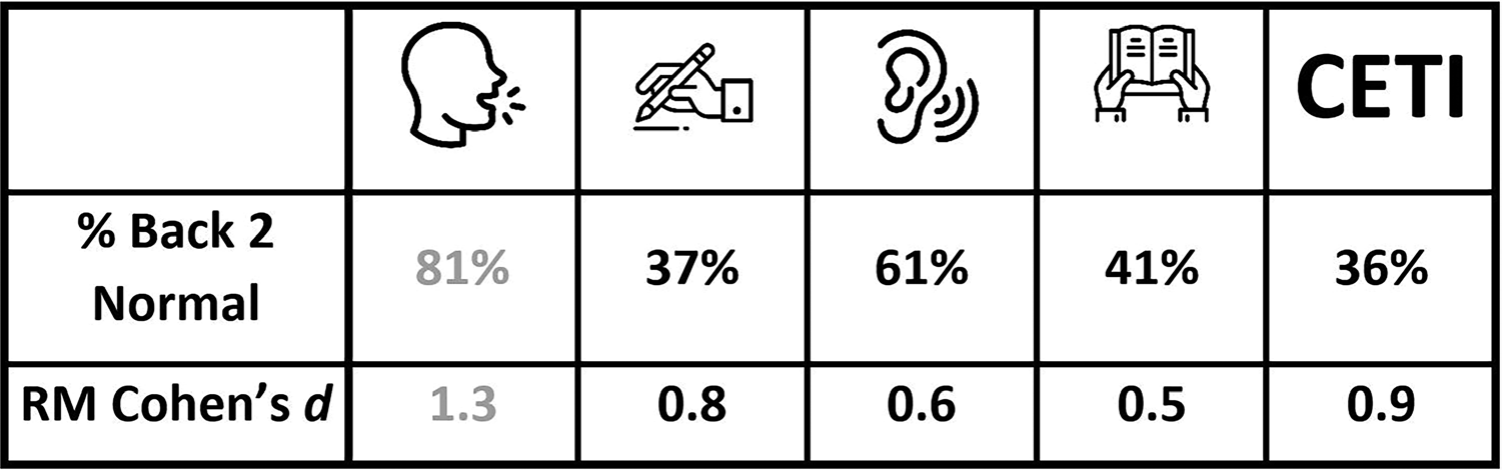
Unstandardized (% back 2 normal) and standardized (repeated measures Cohen’s *d*) for the 4 language domains (speaking, writing, listening, reading) and the functional outcome (Communicative Effectiveness Index [CETI]).

Both unstandardized and standardized scores demonstrated large effect sizes (>0.8 Cohen’s *d*) for language production domains and the CETI, and medium effect sizes (>0.5 Cohen’s *d*) for language perception domains. Speech production improved more than the other language domains (Figure 2).

## Discussion

PWA caused by stroke and who participated in the ICAP made, on average, large gains on both impairment-based and functional measures of language. Gains were sustained and in some cases continued to improve at follow-up. Given the therapeutic effects of the Queen Square ICAP, how does it compare with the other ICAPs? Of the 6 published ICAPs measuring change in language ability, 5 have shown significant improvements, with 2 showing large effect sizes similar to this study.^11,12^ No other published ICAP studies have investigated therapy effects across language domains. Our finding of significantly greater effects on speech production may reflect that this is generally the domain that patients, especially in the chronic stage poststroke, care about and thus wish to work on most. Importantly, the presence of the therapy-by-domain interaction strongly mitigates against either bias or test-retest explanations of our result.

The significant improvements in 2 of the 4 domains at 12 weeks post-ICAP suggests that language gains were not only consolidated but also continued to improve. A similar carry-over effect was seen in the data from the Queen Square upper-limb programme^6^ and one other ICAP.^13^ The CETI scores support this with clinically significant changes (>11.4^8^) in functional communication abilities similar to those reported in one of the other ICAP studies.^11^

Related to this longer-term boost in function is the inclusion of neuropsychological input in our ICAP. Systemic therapy in this context has been shown to work^14^ and may well be a key factor in the long-term effectiveness of our ICAP; as was the interdisciplinary focus on developing self-management skills. Future analysis of PWA and carer mood and quality of life outcomes collected as part of our service will investigate this further.

## Conclusions

Given the effectiveness of this and other ICAPs, why are they not more widely available or considered as part of standard clinical practice? They certainly are resource and staff intensive, and cost is an issue. The Queen Square ICAP has running costs of ≈£300 000 pa which works out at £5500 per patient. This is much more than is currently spent on PWA in the United Kingdom, but it is only about the same as the cost of a hip replacement on the NHS. Rather than the current pragmatic state of service provision for PWA where, at least in the United Kingdom, woefully insufficient resources are shared out equally or in the United States, where resources are concentrated almost exclusively in the acute stage poststroke, should not the available evidence for clinical efficacy dictate how services should be set-up to benefit patients? Given that stroke prevalence is increasing and the compelling evidence we provide here on the effectiveness of an ICAP for chronic PWA, surely now is the time to provide these high-quality services and change lives.

## Sources of Funding

The Intensive Comprehensive Aphasia Program is supported by the National Brain Appeal and the Tavistock Trust for Aphasia. Dr Crinion by Wellcome (106161/Z/14/Z) and Dr Leff by National Institute of Health Research (RP-2015-06-012).

## Disclosures

None.

## Supplemental Materials

The TIDieR guideline template for the Queen Square ICAP

## Supplementary Material


