# Supplementary material for: Clinical Effectiveness of the Queen Square Intensive Comprehensive Aphasia Service for Patients With Poststroke Aphasia
Source: Stroke. 2021 Jun 10;52(10):e594–8. doi: 10.1161/STROKEAHA.120.033837 (PMC8478085; doi:10.1161/STROKEAHA.120.033837)
Supplement: Supplementary file 1 [file str-52-e594-s001.pdf]

SUPPLIMENTARY MATERIAL

A. The TIDieR guideline template for the Queen Square ICAP:

1.	Brief name	The Queen Square Intensive Comprehensive Aphasia service (ICAP)
2.	Why	In 2015-2016, people with aphasia received an average 3.6 hours of Speech and Language Therapy as an inpatient and a further 6.3 hours in the community (Fulop et al. Health Services Delivery Research 2019;7(7), Palmer et al. PloS one. 2018;13(7)). Evidence indicates an average of 98.4 hours of Speech and Language therapy (SLT) is required for positive functional communication outcomes (Bhogal et al. Stroke. 2003;34:987).<br><br>The Queen Square ICAP aims to address this disparity by providing around 85.5 hours of direct Speech and Language Therapy and 10 hours direct Neuropsychology over a three week period. This intervention block is then followed by 3 hour long SLT reviews at 3, 6, and 12 months in addition to email support and onward referrals throughout the 12 month period.<br><br>The Queen Square ICAP adopts a patient-centred goal driven approach which simultaneously addresses all aspects of the World Health Organisation's International Classification of Functioning (WHO ICF) to generate functional, measurable changes to the person with aphasia's (PwA) communication skills and psychosocial wellbeing (WHO 2002). Simultaneously addressing all aspects of the WHO-ICF has been found to produce the best outcomes (Rose et al. Topics in Stroke Rehabilitation. 2013;20(5):379).

3.	Physical and informational materials	-Personal and functional materials for intervention sessions.<br><br>-Tablets loaded with: Tactus Language, Tactus Advanced Language bundles, Tactus Conversation bundle, Listen-In (UCL), iReadMore (UCL).<br><br>-Computers for word processing and internet related participation and impairment level work.<br><br>-Session plans and handouts.

4.	What (procedures)	Comprehensive interdisciplinary intervention is tailored to the PwA's personal goals, profile of aphasia and cognition (as assessed by the CAT, neuropsychological assessment and informal assessment including discussions with family and friends) and the PwA's psychosocial needs, resources and priorities.<br><br>**SPEECH AND LANGUAGE THERAPY INTERVENTIONS**<br>**<u>Interventions for speech comprehension</u>**<br>**Single Word Comprehension Interventions**<br>• Semantic feature analysis (Boyle et al American Journal of Speech-Language Pathology. 1995;4:94, Efstratiadou et al. Journal of Speech, Language, and Hearing Research. 2018;61:1).<br>• Semantic links (Bigland et al. Semantic Links. 1992)<br>• Auditory discrimination tasks with minimal pairs<br>• Auditory word to picture matching<br>• Barrier games using functional words<br><br>**Sentence Level Comprehension Interventions**<br>• Set sentence frames where one content word is altered (sentence level adaptation of minimal pairs at single word level)<br>• Mapping therapy approaches targeting comprehension (Schwartz et al. Aphasiology. 1994;8(1):19)<br>• Sentence judgement tasks<br>• Verb Network Strengthening Treatment (VNeST) (Edmonds et al. American Journal of Speech-Language Pathology. 2011;20:131)<br><br>**Functional / Participation Level Comprehension**<br>To practice and apply the evidence-based approaches described above, patients carried out functional/participation tasks such as:<br><br>• Therapy Groups *(see Groups section below)*<br>• Compensatory strategies such as note taking<br>• Following instructions<br>• Listening to presentations / monologues and asking questions

		• Watching video clips and summarizing back to demonstrate comprehension<br>• Obtaining information from a stranger (e.g. staff at the British Museum) and demonstrating understanding<br>• Practicing phone calls with an emphasis on comprehension<br><br>**<u>Interventions for expressive language</u>**<br>**Single Word Expression Interventions**<br>Single word intervention initially targets functional word lists generated through goal setting and conversations with the PwA, their friends and family before moving on to more generic functional and high frequency targets.<br><br>• Speech sounds on cue (with functional whole words as the target) (Bishop. Speech Sounds on Cue. 2011)<br>• Articulatory kinematic approaches including: phonetic derivation, position derivation, tactile/kinaesthetic feedback on placement, phonological component analysis, modelling, repetition, articulatory placement cues, shaping of place, manner or voice errors, biofeedback using mirrors and video<br>• Intersystemic reorganisation approaches including: iconic gestures for functional words, sentence completion, externally applied rhythm from SLT, choral approaches<br>• Rate and rhythm approaches including: metrical pacing therapy, finger tapping, melodic intonation therapy (Meulen et al. Archives of Physical Medicine and Rehabilitation. 2012;93:S46) and pacing boards at the syllable level<br>• Semantic approaches: semantic feature analysis with semantic distinctions (Boyle 1995 op. cit., Efstratiadou 2018 op. cit.) semantic-phonological word maps (incorporating semantic feature analysis and phonological component analysis based on the individual loci of breakdown)<br>• Phonological and semantic cueing hierarchies (Linebaugh et al. Aphasiology. 2005;19(1):77)

<table>
<tr>
<td></td>
<td></td>
<td>

Barrier games using functional words e.g. Promoting Aphasic’s Communicative Effectiveness (PACE) (Davis, Aphasiology. 2005;19(1):21, Edelman. Promoting Aphasics Communicative Effectiveness 1987)
Compensatory strategies such as total communication, drawing (Byng et al. International Journal of Language and Communication Disorders. 1999;34:265, Sacchett, et al. in The Aphasia Therapy File Volume 2 2007), and gestural facilitation (Raymer et al. Neuropsychological Rehabilitation. 2011;22:235)
Word finding strategies such as circumlocution and self-cueing.

Sentence Level Expression Interventions

Response elaboration training (RET) (Wambaugh et al. American Journal of Speech-Language Pathology. 2013;22(2):S409)
Verb Network Strengthening Treatment (VNeST) (Edmonds op. cit. 2011)
Rate and rhythm approaches including: metrical pacing therapy, finger tapping, melodic intonation therapy and pacing boards at the syllable level
Script training (Goldberg et al. American Journal of Speech-Language Pathology. 2012;21:222)
Mapping therapy (production) (Rochon et al. Neuropsychological Rehabilitation. 2005;15(1):1, Schwartz op.cit. 1994)
Treatment of Underlying Forms (Thompson et al. Aphasiology. 1993;7(1):111)

Discourse Level Expression Interventions

Structured mind mapping approaches for expository narratives highlighting “beginning, middle and end” such as that described by (Whitworth et al. Aphasiology. 2015;29(11):1345)
Procedural narratives
Modified response elaboration treatment for personal recounts (Wambaugh op. cit. 2013)

</td>
</tr>
</table>

		**Function / Participation Level Expressive Language Interventions**<br>To practice and apply the evidence-based approaches described above, patients carried out functional/participation tasks such as:<br>• Therapy Groups such as total communication group (Lawson in Byng et al. The Aphasia Therapy File Volume 1. 1999) and discussions using compensatory strategies including drawing, circumlocution, first letter prompting and gesture (Byng op. cit. 1999; Sacchett op. cit. 2007, Simmons-mackie ey al. Aphasiology. 1997;11(8):761)<br>• Paired interviews<br>• Practical tasks such as ordering coffee, requesting information and making phone calls<br>• Delivering presentations<br>• Debates and discussions<br>• Conversation Partner Training (as described below)<br><br>**<u>Interventions for Reading</u>**<br>**Single word reading interventions**<br>• **Letter by letter reading** using cross modality cueing (e.g. saying the letter sound, letter name and copying) (Nitzberg Lott et al. Aphasiology. 1994;8(2):181)<br>• **"Sounding out" techniques** such as re-learning letter-sound correspondence (GPCs)(Partz. Cognitive Neuropsychology. 1986;3(2):149)<br>• **Whole word recognition tasks** e.g. single written word to picture matching, or recognition and reading of irregular words (Coltheart in Seron et al. Cognitive Approaches to Neuropsychological Rehabilitation 1989)<br>• **Homophone judgment / identification** (Scott et al. Aphasiology. 1989;3(3):301)<br><br>**Sentence / paragraph / text level reading**<br>• **Repetitive reading aloud** at sentence level to improve familiarity and comprehension such as **Multiple Oral Reading (MOR)** (Kim. Contemporary Issues in Communication Science and Disorders. 2010;37,

		Tuomainen et al. Aphasiology. 1991;5:401) and **Oral Reading for Language in Aphasia (ORLA)** (Cherney et al. Rehabilitation Literature. 1986;47:112)<br>• **Strategy based treatments** such as using a **place keeper, focused attention, reading and re-reading.** (Cocks et al. Aphasiology. 2013;27:509, Gold et al. Reading Psychology. 1984;5:65, Lynch et al. Asia Pacific Journal of Speech, Language and Hearing. 2009;12(3):221, Webster et al. Aphasiology. 2013;27(11):1362)<br>• **Reading & Summarizing tasks** such as **Attentive reading and constrained summarization (ARCS)** (Rogalski et al. Aphasiology. 2008;22:763)<br>• **VneSt** (Edmonds op. cit. 2011)<br>• **Reading for inference** (Baretta et al. Psychology & Neuroscience. 2009;2:137)<br>• **Supported reading comprehension** - incorporating aphasia-friendly text supports such as **pictures or underlining key words** (Dietz et al. Aphasiology. 2009;23:1053, Knollman-Porter et al. Topics in Stroke Rehabilitation. 2016;23(4), Rose et al. Aphasiology. 2003;17(10),:947)<br>• **Compensatory strategies such as using text to speech apps** (Harvey et al. Communication Disorders Quarterly. 2013;35)<br><br>**Functional / Participation Reading tasks**<br>To practice and apply the evidence-based approaches described above, patients carried out functional reading tasks such as:<br>• Sending text messages and emails<br>• Reading headlines and matching to pictures or text<br>• Reading articles or fiction (with re-reading), summarizing and discussing as a group<br>• Reading aloud children's story as part of MOR approach<br>• Reading menus and ordering as part of a constraint induced approach.

		• Reading important documents such as appointment letters and highlighting the key information.<br><br>**<u>Interventions for Writing</u>**<br>**Single word interventions for writing**<br>• **Phoneme-Grapheme Re-Training** (Hillis Trupe in Brookshire. Clinical Aphasiology. 1986, Kiran. Aphasiology. 2005;19(1):53)<br>• **Mapping Sounds to letters using a key word** (Beeson et al. Aphasiology. 2000;14:551)<br>• **Written Naming Tasks** (Hillis et al. Brain and Language 1991;40(1):106)<br>• **Anagram and Copy Treatment** (ACT) (Beeson et al. Aphasiology. 2002;16:473)<br>• **Copy and Recall Treatment** (CART) (Beeson et al. Journal of Speech, Language, and Hearing Research. 2003;46(5):1038)<br>• **Self-dictation strategy** (Pound. Aphasiology. 1996;10(3):283)<br><br>**Sentence / paragraph / text level writing**<br>• **<u>VneSt</u>** (Edmonds op. cit. 2011)<br>• **Written picture description using sentence verb object structure** (Salis et al. Aphasiology. 2010;24(9):1051)<br>• **Compensatory strategies such voice recognition or "speech to text"** (Bruce et al. International Journal of Language & Communication Disorders. 2003;38:13, Estes et al. Aphasiology. 2011;25(3):366, Thiel et al. Aphasiology. 2015;29(4):423)<br>• **Answering Wh-questions using a modified Treatment of Underlying Forms approach** (Thompson op. cit. 1993)<br>• **Sentence dictation tasks**<br>• **Metacognitive approach** – writing discourse after analyzing example texts and identifying "beginning, middle, end" – such as the narrative therapy approach described in (Whitworth op. cit. 2015)<br><br>**Function/participation level tasks for writing**

		To practice and apply the evidence-based approaches described above, patients carried out functional writing tasks such as:<br><br>• Writing cards / notes<br>• Texting<br>• Writing emails<br>• Structured writing tasks such as filling an application, writing a CV<br>• Writing presentations with scaffolding<br>• Maintaining an aphasia diary / journal / written reflection<br>• Creative writing tasks – writing a short story based after rearranging pictures.<br><br>**Computer/ Apps**<br>The following multi-modal apps were used at single word level, sentence level and discourse level. These were used to treat comprehension, expression, reading and writing.<br><br>• Tactus Language Therapy 4-in-1 (Tactus Therapy Solutions Ltd., 2011)<br>• Tactus Advanced Language Therapy 4-in-1 (Tactus Therapy Solutions Ltd., 2018)<br>• Cue Speak (Cue Speak ltd, 2020)<br>• Speech Sounds on Cue (Bishop op. cit. 2011)<br>• React 2 (React2 Ltd., 2005)<br>• Listen-In (Fleming et al. Journal of Neurology, Neurosurgery & Psychiatry. 2021;92(4):418)<br>• iReadMore (Woodhead et al. Brain. 2018;141(7):2127)<br><br>**Groups and participation level interventions**<br>• Total communication (Lawson op. cit. 1999)<br>• Conversation partner training with friends, carers and or family involves using video for PWA and their family/friends to identify the most effective strategies to support communication (Simmons-Mackie et al. Arch Phys Med Rehabil. 2016;97(12):2202)<br>• Aphasia education<br>• “Explaining my aphasia” and self advocacy

		• Discussion group<br>• Newspaper group<br>• Metacognitive strategies tailored to the individual eg for auditory breakdown this may be "pre listening- task listening – post listening"<br>• Scripting and practicing person centered and goal related tasks<br>• Community tasks<br>• Supported liaison with place of work or education<br>• AAC<br><br>**NEUROPSYCHOLOGY INTERVENTIONS**<br>**<u>Group interventions</u>**<br>**Narrative therapy group**<br>Tree of Life narrative therapy framework (Ncube. International Journal of Narrative Therapy & Community Work. 2006;(1):3) used to support individuals to:<br>• Share and reflect on collective experience of living with aphasia<br>• Develop a strengths-based narrative<br>• Identify personal and social resources to help them achieve and sustain preferred future.<br><br>**Family and friends support group**<br>Psychotherapeutic support group focusing on adaptation to major life changes, self-management and emotional wellbeing (Off et al. Topics in Language Disorders. 2019;39(1):5)<br><br>**Couple/family therapy interventions**<br>(Nichols et al. Aphasiology. 1996;10(8):767, Meredith. Psychotherapy and Aphasia: Interventions for Emotional Wellbeing and Relationships. 2019)<br>• Genogram to elicit information about family beliefs, attitudes and impact of aphasia on family life cycle<br>• Externalising questions to identify influence of aphasia on family and influence of family on aphasia-related problems<br>• Circular questions to identify patterns of behavior that maintain/diminish problem

		• Unique outcome questions to explore strengths and resources of family members that they can use to support each other<br><br>**<u>Individual interventions</u>**<br>**Behaviour therapy**<br>(Thomas et al. Clinical rehabilitation. 2013;27(5):398)<br>• Graded goal hierarchy<br>• Activity scheduling<br>• Relaxation training<br>• Graded exposure<br><br>**Cognitive behavior therapy**<br>(Kneebone. Cognitive and Behavioral Practice. 2016;23(1);99)<br>As above, plus:<br>• Cognitive restructuring, using behavioural experiments, surveys, cost and benefit analyses<br>• Relapse prevention / self-management<br><br>**Solution-focused brief therapy**<br>(Northcott et al. Folia Phoniatrica Logopedica. 2016;67(3):156)<br>• Description of preferred future<br>• Scaling questions<br><br>**NEUROLOGY INTERVENTION**<br>One 'meet the doctor' session per cohort with the consultant neurologist. A 1 hour open discussion/psychoeducational session on a variety of topics relating to stroke (e.g. causes, prognosis and prevention) and rehabilitation (e.g. recovery trajectories, plasticity).

5.	Who (provider)	2.0 Trained Highly Specialist Speech and Language Therapists (SLT) with a special interest in Aphasia<br><br>2.0 Speech and Language Therapy Assistants trained and supervised by the SLTs.<br><br>0.5 WTE Clinical Neuropsychologist for all group and individual interventions, supported by 0.1 wte Trainee Clinical Psychologist for group intervention with PwA.<br><br>0.1 WTE consultant neurologist
6.	How	SLT intervention delivered in the following formats:<br>-Daily direct one to one sessions (typically 1-1.5 hours in length)<br>-Paired direct sessions<br>-Direct sessions with the PwA's friends and or family (where this is not possible this session will be over the phone)<br>-Groups sessions<br>-Independent work eg supervised app time/project related tasks<br><br>-Unsupervised independent tasks
7.	Location	Therapy rooms in the hospital<br>In the community
8.	How much	The PwA attended the hospital weekdays from 9 am until 4:30 pm.<br><br>They received 85.5 hours SLT and 9 hours Neuropsychology within the three week period.<br><br>The PwA then received a further average of 1.5 hours direct 1:1 intervention with the SLT at 3, 6 and 12 months.
9.	Tailoring	Tailored communication and cognitive supports are developed based on Speech and Language Therapy and Psychology recommendations for maximising client engagement with therapeutic interventions (e.g. facilitator drawing or writing on

		behalf of client, or using written options to facilitate verbal generation).
10.	Modifications	The balance and content of intervention is modified according to the PwA's goals, strengths and weaknesses.
11/12	How well (planned)	The main aim was to get the dose as high as possible on each therapy day with the initial aim of 7.5 hours. This was rarely practicable. We revised this down to around 6 hours a day, but took note of patient fatigue and other factors that meant that we didn't always achieve this dosing level.
