# Supplementary figures and images for: Clinical Effectiveness of the Queen Square Intensive Comprehensive Aphasia Service for Patients With Poststroke Aphasia
Source: Stroke. 2021 Jun 10;52(10):e594–8. doi: 10.1161/STROKEAHA.120.033837 (PMC8478085; doi:10.1161/STROKEAHA.120.033837)

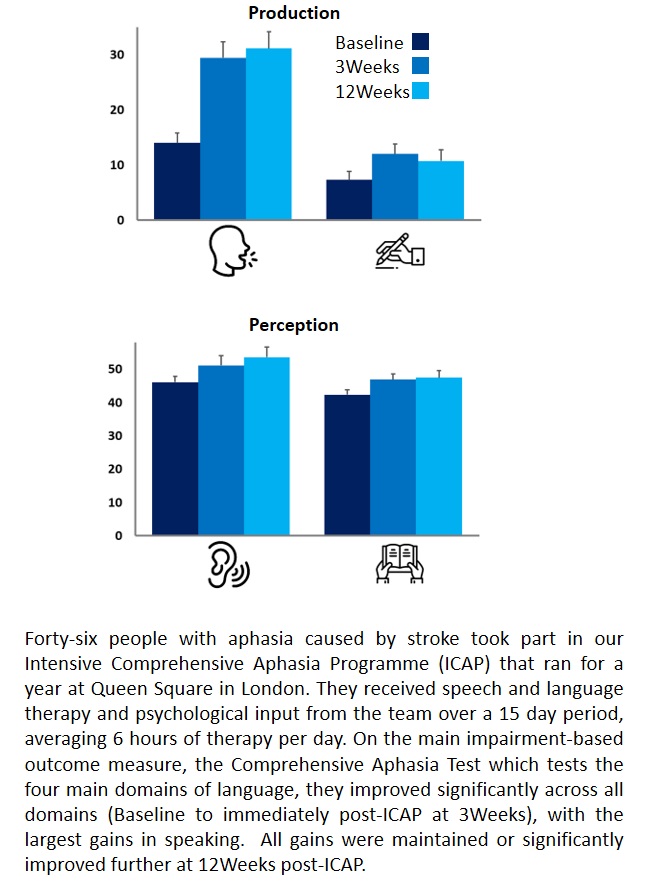

Supplement: Supplementary file 2 [file str-52-e594-s002.jpg]
